# Integrated TCRγ Clonality Workflow for Molecular Diagnosis in HTLV-1 Carriers and Adult T-Cell Leukemia/Lymphoma

**DOI:** 10.3390/diagnostics16142288

**Published:** 2026-07-22

**Authors:** Davi Ferreira Jofre, Giulia Sales Guimarães, Ana Beatriz Rocha, Jéssica Bilar Cavalcanti, Priscila de Lima Barros, Karine Sobral Marques, Nélio Cézar de Aquino, Vinícius de Camargo Callefi, Isadora Alves, Lilian de Souza França, Letícia Montanha de Assis, Sheila de Oliveira Garcia Mateos, Carolina Rosadas, Jorge Simão do Rosário Casseb, Youko Nukui, Vanderson Rocha, Luís Alberto de Pádua Covas Lage, Cadiele Oliana Reichert, Juliana Pereira, Hebert Fabricio Culler

**Affiliations:** 1 Laboratory of Pathogenesis and Directed Therapy in Onco-Immuno-Hematology (LIM-31), Department of Hematology, Hemotherapy, and Cell Therapy, Faculty of Medicine, University of Sao Paulo (FM-USP), Sao Paulo 05419-000, Brazil; davi.jofre@hc.fm.usp.br (D.F.J.); giuliasalesguimaraes@gmail.com (G.S.G.); beatrizrrocha.ana@gmail.com (A.B.R.); jessica98.cavalcanti@gmail.com (J.B.C.); priscila.barros@usp.br (P.d.L.B.); karinesmarques@gmail.com (K.S.M.); nelio.aquino@alumni.usp.br (N.C.d.A.); vinicallefi@gmail.com (V.d.C.C.); isadora.alves26@hotmail.com (I.A.); sf.lilian@hotmail.com (L.d.S.F.); leticia.montanha@hc.fm.usp.br (L.M.d.A.); vanderson.rocha@prosangue.sp.gov.br (V.R.); luis.lage@hc.fm.usp.br (L.A.d.P.C.L.); cadiele@usp.br (C.O.R.); juliana.pereira@hc.fm.usp.br (J.P.); 2 Real-World Evidence & Precision Public Health Team, Laboratory of Pathogenesis and Directed Therapy in Onco-Immuno-Hematology (LIM-31), Department of Hematology, Hemotherapy, and Cell Therapy, Faculty of Medicine, University of Sao Paulo, Sao Paulo 05419-000, Brazil; 3 Brazilian Health Regulatory Agency (Agência Nacional de Vigilância Sanitária—ANVISA), Brasilia 71205-050, Brazil; 4 Department of Research and Innovation, Campus Medicine, Municipal University of Sao Caetano do Sul, Sao Caetano 09521-160, Brazil; sheila.mateos@online.uscs.edu.br; 5 Section of Virology, Department of Infectious Diseases, Faculty of Medicine, Imperial College, London W2 1PG, UK; c.rosadas-de-oliveira@imperial.ac.uk; 6 Laboratory of Medical Investigation in Dermatology and Immunodeficiencies (LIM-56), Instituto de Medicina Tropical (IMT), Faculty of Medicine, University of São Paulo, Sao Paulo 05403-907, Brazil; jorge.casseb@hc.fm.usp.br; 7 Clinical Hospital of the University of Sao Paulo Medical School (HC-FMUSP), Sao Paulo 05419-000, Brazil; youko.nukui@hc.fm.usp.br

**Keywords:** T-cell receptor gamma, clonality, HTLV-1, adult T-cell leukemia/lymphoma, multiplex PCR, molecular diagnostics, EuroClonality

## Abstract

**Background/Objectives**: T-cell receptor gamma (TCRγ) clonality testing by multiplex polymerase chain reaction (PCR) is an important ancillary method in the diagnostic evaluation of T-cell lymphoproliferative disorders. Interpretation is particularly challenging in HTLV-1 infection, in which persistent antigenic stimulation may produce clonal or oligoclonal T-cell expansions overlapping with non-neoplastic patterns. This study aimed to assess the diagnostic performance and interpretative contribution of two EuroClonality/BIOMED-2 assays (TCRγ-A and TCRγ-B), considered complementary components of the same analytical panel, and a single-tube multiplex PCR assay (TCRγ-O), and to develop an integrated workflow for TCRγ clonality interpretation. **Methods**: A total of 107 peripheral blood samples were analyzed, including 19 healthy donors, 75 HTLV-1 carriers, and 13 patients with adult T-cell leukemia/lymphoma (ATLL). TCRγ clonality was evaluated using three multiplex PCR assays followed by capillary electrophoresis. Profiles were classified as polyclonal, oligoclonal, or monoclonal. Diagnostic performance analysis was performed using healthy donors as the reference negative group and ATLL patients as the reference positive group. **Results**: The combined EuroClonality approach (TCRγ-A+B) showed the highest sensitivity (100%), whereas TCRγ-O showed the highest specificity (94.7%). TCRγ-A and TCRγ-B each showed a sensitivity of 76.9%, with specificities of 84.2% and 78.9%, respectively. Classification was highly concordant in healthy donors and ATLL patients employing both assays. However, among HTLV-1 carriers, classification varied across the assays. The integrated workflow was associated with a significant change in the distribution of molecular classification patterns in the HTLV-1 group compared with EuroClonality-based interpretation alone. (Stuart–Maxwell, *p* < 0.001). **Conclusions**: The EuroClonality/BIOMED-2 strategy provided greater sensitivity, whereas TCRγ-O showed greater specificity. Their integration supported a complementary and hierarchical approach to TCRγ clonality assessment, particularly in HTLV-1 infection. These findings support an exploratory integrated framework for TCRγ clonality interpretation in HTLV-1 infection and ATLL, requiring validation in larger independent cohorts.

## 1. Introduction

Molecular clonality testing has become an important ancillary tool in the diagnostic work-up of suspected T-cell lymphoproliferative disorders [1,2]. In routine practice, the demonstration of a clonal T-cell population may provide strong support for the presence of a neoplastic process; however, clonality results must always be interpreted in conjunction with clinical, morphologic, and immunophenotypic findings, because clonal or oligoclonal T-cell expansions may also occur in non-neoplastic settings [3,4,5,6]. For this reason, current recommendations emphasize that T-cell receptor (TCR) gene rearrangement analysis is not a stand-alone diagnostic test, but rather a complementary method whose value depends on careful technical performance and contextual interpretation [4,7,8].

Among the available molecular targets for T-cell clonality assessment, the TCR gamma (TCRγ) locus has practical relevance [9]. In comparison with other TCR targets, TCRγ rearrangements are especially suitable for PCR-based analysis because of the relatively restricted repertoire of rearrangements, the high frequency of TCRγ locus rearrangement in clonal T-cell populations, and the applicability of this target to both αβ and γδ T-cell clones [10,11,12]. These characteristics have made TCRγ a widely used target in multiplex PCR assays followed by capillary electrophoresis, including the standardized EuroClonality/BIOMED-2 approach, which remains a major reference framework for diagnostic clonality testing in lymphoproliferative disorders [4,7,12].

The biological basis of clonality testing lies in variable, diversity, and joining (V(D)J) recombination, the somatic process through which TCR genes are assembled during T-cell development [4,13,14,15]. This mechanism generates a highly diverse antigen receptor repertoire and is essential for adaptive immune recognition [4,15,16]. Under physiological conditions, such diversity is reflected in polyclonal molecular profiles [4,16,17]. By contrast, skewed repertoires, oligoclonal expansions, or dominant clonal peaks may emerge when T-cell populations undergo selective expansion. Importantly, these patterns are not exclusive to malignancies [16,17,18]. They may also be observed in chronic antigenic stimulation, immune dysregulation, immunosenescence, immunodeficiency, or reactive proliferations [5,18,19], which explains why interpretation of molecular clonality requires a clinicopathologic framework rather than a purely binary molecular reading [2,17].

This interpretive challenge is particularly relevant in the setting of human T-lymphotropic virus type 1 (HTLV-1) infection [20,21,22]. HTLV-1 establishes persistent infection in T cells and promotes long-term expansion of infected cellular clones [20,23,24,25]. As a result, infected individuals may exhibit a spectrum of clonal architectures ranging from broad polyclonality to marked oligoclonal or dominant clonal expansions [21,23,26]. This biological continuum complicates the distinction between reactive or virus-associated proliferative states and overt malignant transformation [20,23,27,28]. Recent work on clonal dynamics in HTLV-1 infection has shown that the size and distribution of infected clones may carry prognostic significance, and that clonality-related features are relevant to the risk of progression to adult T-cell leukemia/lymphoma (ATLL) [23,26,27,29].

In this setting, oligoclonal profiles should not be regarded simply as technical ambiguity. Rather, they may reflect a biologically intermediate state resulting from chronic viral persistence, antigen-driven immune responses, expansion of HTLV-1-infected clones, or early clonal selection preceding overt malignant transformation [20,21,22,23,26,27]. Therefore, the interpretation of oligoclonality in HTLV-1 carriers requires particular caution, because such patterns may represent either non-malignant virus-associated immune dynamics or early clonal evolution along the pathway toward ATLL [21,23,27,29,30].

ATLL represents the leukemic/lymphomatous neoplasm classically associated with HTLV-1 infection and is recognized as a mature T-cell neoplasm in current hematolymphoid classifications [1,31,32,33,34]. From a biological standpoint, a greater degree of clonal dominance is expected in ATLL than in asymptomatic carriers or other non-neoplastic contexts, although the interpretation of any single clonality assay remains conditioned by assay design, technical sensitivity, and the complexity of the underlying T-cell population. Therefore, the comparison of healthy donors, HTLV-1 carriers, and patients with ATLL provides a particularly informative biological model in which to examine the analytical and interpretative behavior of TCRγ clonality assays across a gradient that extends from physiological polyclonality to overt neoplasia [21,26,35].

The EuroClonality/BIOMED-2 system has been one of the most influential standardization efforts in immunoglobulin/T-cell receptor (Ig/TCR) clonality testing. Its diagnostic value lies not only in primer design and analytical coverage, but also in its emphasis on harmonized interpretation and reporting [4,7]. Within this framework, the TCRγ-A and TCRγ-B reactions were conceived as complementary tubes within the same analytical set rather than as independent competing assays. Their combined use broadens coverage of relevant rearrangements and may improve sensitivity for detecting clonal populations [4,7,36]. At the same time, other single-tube multiplex PCR strategies for TCRγ analysis have been described and may display different analytical profiles because of differences in primer composition, target coverage, and electropherogram patterning REF [36,37,38]. Consequently, discrepancies between assays are biologically and technically plausible, especially in samples with low-level or complex clonal expansions [4,12,36,39].

The theoretical basis for the proposed diagnostic strategy is that TCRγ clonality detection is strongly influenced by primer design, target coverage, tube architecture, and fragment interpretation. Within the EuroClonality/BIOMED-2 system, TCRγ-A and TCRγ-B are not redundant reactions, but complementary tubes designed to interrogate different Vγ gene segment groups within the TCRγ locus, while sharing joining-region targets and standardized interpretative principles. This distribution reduces the likelihood that a clonal rearrangement will be missed because of incomplete primer coverage, preferential amplification, primer-template mismatch, or restricted rearrangement usage [4,7,12]. In contrast, the single-tube TCRγ-O assay applies a different multiplex configuration, simultaneously targeting multiple Vγ and Jγ combinations in a single reaction, which may generate a distinct electropherographic readout and provide an independent interpretative layer [35,36,37,38]. Therefore, the complementarity of these assays does not rely only on repeating the same molecular question, but on combining partially distinct primer architectures and analytical behaviors to improve the balance between sensitivity, specificity, and interpretative confidence [4,7,17,36,37,38,39].

In this context, the key question is not simply which assay is better in absolute terms, but how distinct assays behave when applied to biologically different groups and whether their integration can improve the interpretative consistency of T-cell clonality assessment [4,17]. This issue is especially important in HTLV-1 infection, in which borderline, oligoclonal, and weakly dominant patterns may be encountered more frequently than in unequivocally negative or frankly neoplastic samples [23,40,41,42]. A strategy that privileges screening sensitivity alone may overlook biologically ambiguous patterns, whereas a more conservative assay may improve specificity at the cost of reduced detection of subtle clonal expansions. An integrated diagnostic approach may therefore offer a more balanced interpretation than reliance on a single molecular result [4,12,42].

Therefore, this study aimed to compare the diagnostic performance and interpretative behavior of EuroClonality/BIOMED-2 TCRγ-A/TCRγ-B assays and a complementary single-tube TCRγ-O assay in healthy donors, HTLV-1 asymptomatic carriers, and ATLL patients. We further developed an exploratory integrated interpretative workflow to assess whether the combined interpretation of complementary TCRγ assays could refine the classification of non-clonal, oligoclonal, and clonal profiles in a biologically heterogeneous setting.

## 2. Materials and Methods

### 2.1. Study Design and Study Population

This analytical observational study was based on peripheral blood samples obtained from adults followed at the HTLV-1 Outpatient Clinic of the Discipline of Hematology, Hemotherapy and Cellular Therapy, Hospital das Clínicas, Faculty of Medicine, University of Sao Paulo (HC-FMUSP), São Paulo, Brazil. The source population comprised asymptomatic HTLV-1 carriers with a defined route of viral transmission and patients with adult T-cell leukemia/lymphoma (ATLL), including indolent and aggressive clinical forms according to the Shimoyama classification system [43], who were under follow-up at the institution. Healthy controls were recruited from platelet apheresis donors at Pro-Sangue Foundation, Blood Bank of Sao Paulo, following Consolidation Ordinance No. 5, of 28 September 2017, of the Brazilian Ministry of Health recommendations [44]. Only samples from individuals with two plateletpheresis donations were included, because repeated plateletpheresis may reduce cluster of differentiation 4-positive (CD4+) T-cell counts [45].

Eligible participants were adults aged over 18 years. HTLV-1 infection was confirmed by two polymerase chain reaction assays performed on samples collected at different time points before recruitment. In the source population, asymptomatic HTLV-1 carriers were defined as HTLV-1-infected individuals without clinical, morphologic, or immunophenotypic evidence of ATLL at the time of inclusion. ATLL diagnosis was established in HTLV-1-positive individuals using a composite clinicopathological approach, according to the Levine Diagnostic Criteria [46], and cases were subsequently classified according to the Shimoyama criteria [43]. Diagnosis was not based on a single laboratory parameter, but on the integration of clinical presentation, hematological findings, cytomorphology, immunophenotypic evidence of abnormal/clonal T-cell expansion by flow cytometry, and/or compatible tissue biopsy when available [43,46]. Flow cytometric assessment of clonal or aberrant T-cell expansion was performed as part of the institutional diagnostic work-up in an independent laboratory, separately from the TCRγ assays evaluated in the present study. Therefore, the TCRγ results analyzed here were interpreted as molecular correlates of the established clinicopathological diagnosis and as complementary evidence supporting the immunophenotypic findings, rather than as the sole criterion defining ATLL case status. Exclusion criteria in the source population included inability to define the route of HTLV-1 transmission, concomitant acute or chronic infections, other malignant neoplasms except non-melanoma skin cancer, and primary cutaneous aggressive tumoral ATLL as defined by Bittencourt A et al. [47].

In the source population, asymptomatic HTLV-1 carriers were stratified according to the presumed route of infection as vertical/breastfeeding, sexual, or transfusional, based on medical history, maternal serology, partner serology, and transfusion history. Classification of vertical transmission, presumed to have occurred through vertical/breastfeeding, required maternal anti-HTLV I/II positivity, negative anti-HTLV I/II serology in all current and previous sexual partners, and no history of transfusion of blood components before November 1993, when anti-HTLV I/II screening became mandatory in Brazilian blood banks under Ordinance No. 1376/1993 of the Brazilian Ministry of Health [48]. Classification of sexual transmission required negative maternal anti-HTLV I/II serology, anti-HTLV I/II positivity in a current and/or previous sexual partner, and no history of transfusion of blood components before November 1993. For both transmission routes, participants were required to report no previous intravenous drug use involving needle sharing. In the present study, no participants were classified as having acquired HTLV infection through the transfusional route.

**Figure 1:**
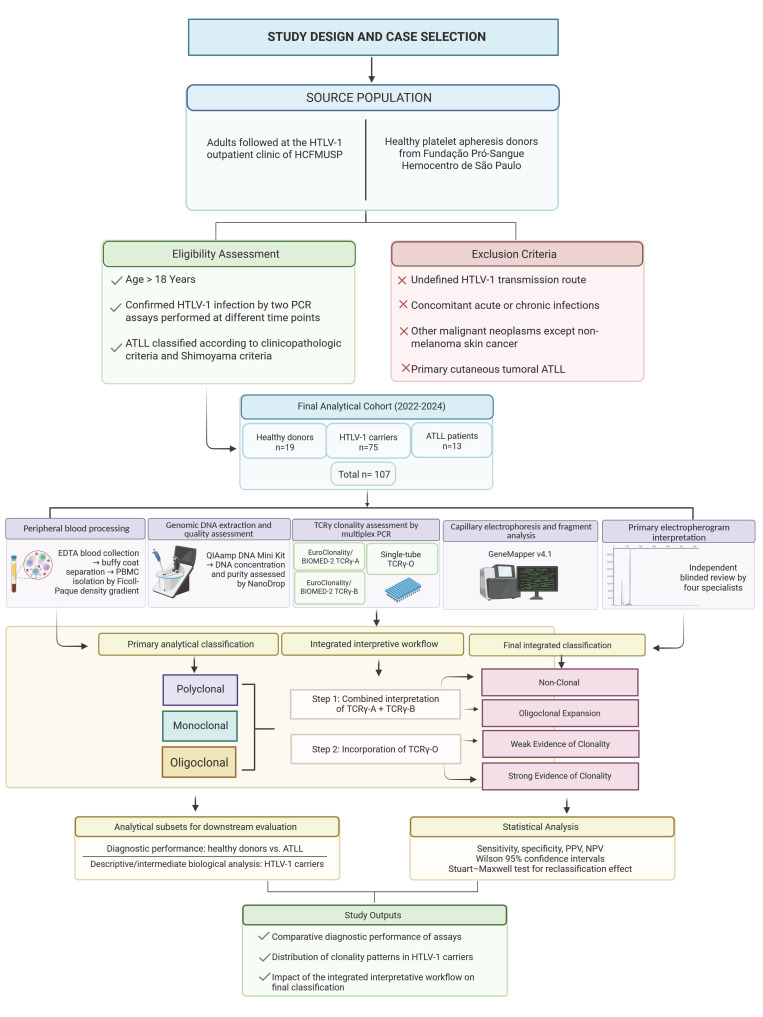
Methodological flowchart of the study. Peripheral blood samples from healthy donors, HTLV-1 carriers, and ATLL patients were used for peripheral blood mononuclear cell (PBMC) isolation and genomic DNA extraction. TCRγ clonality was analyzed with EuroClonality/BIOMED-2 assays (TCRγ-A, TCRγ-B) and a single-tube assay (TCRγ-O), followed by capillary electrophoresis and blinded review. Profiles were classified as polyclonal, oligoclonal, or monoclonal and integrated into a hierarchical workflow to yield final categories: non-clonal, oligoclonal expansion, weak clonality, or strong clonality. Diagnostic performance and reclassification were then assessed. Created in: https://BioRender.com.

For the present TCRγ clonality analysis, 107 peripheral blood samples collected between February 2023 and December 2025 were included, comprising 19 healthy controls, 75 HTLV-1 asymptomatic carriers, and 13 patients with ATLL. Among the ATLL cases, 4 were classified as acute, 2 as chronic, 1 as lymphoma, and 6 as smoldering according to Shimoyama subtypes [43]. These three groups were selected to represent physiologic, biologically heterogeneous, and overtly neoplastic conditions, respectively, thereby providing a clinically informative framework for evaluating the analytical and interpretative performance of TCRγ clonality assays across distinct biological states; see Figure 1.

### 2.2. Ethical Approval

This study was approved by the institutional Research Ethics Committee under protocol number 60994322.4.0000.0068. Informed consent was obtained from all subjects involved in the study.

### 2.3. Sample Processing and Genomic DNA Extraction

Peripheral blood samples were collected in ethylenediaminetetraacetic acid (EDTA) containing tubes, and peripheral blood mononuclear cells (PBMCs) were isolated by density gradient centrifugation using Ficoll-Paque Plus (Cytiva, Uppsala, Sweden), according to the manufacturer’s instructions. Genomic DNA was extracted using the QIAamp DNA Mini Kit (QIAGEN, Hilden, Germany). DNA concentration and purity were assessed using a NanoDrop 1000 Spectrophotometer V3.8 (Thermo Fisher Scientific, Waltham, MA, USA). DNA samples were stored at −20 °C until PCR analysis [49].

### 2.4. TCRγ Clonality Analysis by Multiplex PCR

TCRγ clonality was investigated using three multiplex PCR assays: two assays based on the EuroClonality/BIOMED-2 protocol, designated TCRγ-A and TCRγ-B, and one complementary single-tube multiplex PCR assay, designated TCRγ-O. In the present study, TCRγ-A and TCRγ-B were not treated as independent competing assays, but rather as complementary components of the same EuroClonality/BIOMED-2 analytical panel. Their integrated interpretation was considered analytically relevant because the two tubes broaden the coverage of the investigated rearrangements and provide mutual interpretative support. PCRs were performed in a Veriti 96-Well Thermal Cycler (Applied Biosystems, Foster City, CA, USA). Amplified products were subsequently analyzed by capillary electrophoresis, and electropherogram interpretation was performed using Gene Mapper software version 4.1 (Applied Biosystems, Foster City, CA, USA).

### 2.5. Primer Sets, PCR Conditions, and Fragment Analysis

Each multiplex PCR contained 2.5 µL of 10× buffer, 0.75 µL of 50 mM MgCl_2_, 0.5 µL of 10 mM deoxynucleotide triphosphates (dNTPs), 1 µL of a seven-oligonucleotide mix, 0.2 µL (1 U) of Platinum Taq DNA Polymerase, 18.55 µL of ultrapure water, and 1.5 µL of DNA template. The primer sequences were designed according to Shadrach and Warshawsky (2004) [35]. For the assays, an average DNA concentration of 50 ng/µL was used.

The TCRγ-O assay followed a single-tube multiplex design including primers directed against multiple variable gamma (Vγ) region segments (Vγ1–8, Vγ9, Vγ10, and Vγ11) in combination with joining gamma (Jγ) region primers (Jγ1/2, JγP1/2, and JγP). According to the protocol used in this study, amplification consisted of an initial denaturation step of 3 min at 94 °C, followed by 40 cycles of 1 min at 95 °C, 30 s at 61.8 °C, and 30 s at 72 °C, with a final extension of 10 min at 72 °C and holding at 4 °C. Fragment interpretation was performed within the range of 150 to 250 base pairs (bp) using 6-carboxyfluorescein (FAM) and hexachloro-fluorescein (HEX) labeling, as described in Table 1.

The EuroClonality/BIOMED-2 TCRγ-A and TCRγ-B assays followed a standardized two-tube multiplex design [7]. The TCRγ-A tube included primers targeting Vγ1f and Vγ10, whereas the TCRγ-B tube included primers targeting Vγ9 and Vγ11; both tubes used joining-region primers directed to Jγ1.1/2.1 (JP1/2) and Jγ1.3/2.3 (Jγ1/2). Amplification consisted of an initial denaturation step of 5 min at 95 °C, followed by 40 cycles of 45 s at 95 °C, 45 s at 60 °C, and 45 s at 72 °C, with a final extension of 10 min at 72 °C and holding at 4 °C. For fragment analysis, the ranges considered were 145–175 bp and 195–230 bp (FAM) and 175–195 bp and 230–255 bp (HEX) for TCRγ-A, and 80–110 bp and 145–170 bp (FAM) and 110–145 bp and 170–210 bp (HEX) for TCRγ-B, as summarized in Table 2. All 5′ probes used in the TCRγ-A and TCRγ-B assays were designed by the authors of the present study and were not derived from the EuroClonality/BIOMED-2 protocol.

The diagnostic strategy integrated three complementary assays characterized by distinct primer annealing sites, target configurations, and fragment-size analysis. The EuroClonality/BIOMED-2 TCRγ multiplex PCR was performed using two separate tubes with non-overlapping annealing regions: tube TCRγ-A contained primers targeting the Vγ1f and Vγ10 segments, whereas tube TCRγ-B targeted Vγ9 and Vγ11. Both reactions utilized specific Jγ1.1/2.1 and Jγ1.3/2.3 joining-region primers [7]. In contrast, the TCRγ-O assay was executed as a single-tube multiplex reaction utilizing an entirely independent primer configuration that simultaneously annealed to Vγ1–8, Vγ9, Vγ10, and Vγ11 segments, combined with Jγ1/2, JγP1/2, and JγP joining-region primers [35]. Thus, although targeting the same locus, the three assays use distinct primer combinations and annealing dynamics, generating independent electropherographic profiles.

### 2.6. Electropherogram Interpretation

Electropherographic profiles generated in Gene Mapper were interpreted into three primary analytical categories: polyclonal, oligoclonal, and monoclonal. Interpretation was based on the overall distribution pattern of amplified fragments within the expected size ranges for each assay, taking into account the configuration of the electropherogram and the presence or absence of dominant discrete peaks. In general, polyclonal profiles were characterized by a broad distribution of fragments without a clearly dominant peak, whereas monoclonal profiles were characterized by a dominant discrete peak or sharply restricted peak pattern compatible with clonal expansion. Oligoclonal profiles were defined as intermediate patterns showing limited peak restriction or multiple prominent peaks that did not support classification as unequivocally polyclonal or monoclonal. The primary analytical interpretation previously adopted in the study comprised these three categories, and the integrated workflow subsequently incorporated this classification into higher-order interpretative groups.

**Figure 2:**
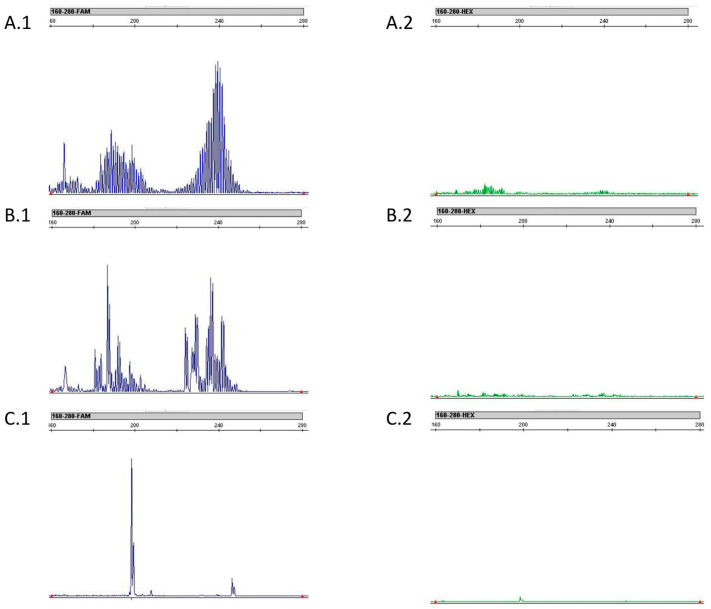
Representative electropherographic profiles obtained with the single-tube T-cell receptor gamma assay (TCRγ-O), illustrating polyclonal (**A.1**,**A.2**), oligoclonal (**B.1**,**B.2**), and monoclonal (**C.1**,**C.2**) patterns. Fragments labeled with 6-carboxyfluorescein (FAM) are shown on the left, and fragments labeled with hexachloro-fluorescein (HEX) are shown on the right.

Electropherogram review was performed independently and in a blinded manner by three different specialists. Discordant interpretations were resolved by consensus after independent primary classification. This primary classification was used for concordance analyses and for comparisons among the three assays. In addition, for selected analyses, results were also evaluated in a binarized form, in which monoclonal patterns were considered clonal, whereas polyclonal and oligoclonal patterns were considered non-clonal, in accordance with the analytical framework adopted in the statistical analysis and with general recommendations for Ig/TCR clonality testing. Representative electropherograms illustrating polyclonal, oligoclonal, and monoclonal profiles in the TCRγ-O assay are shown in Figure 2.

Interobserver agreement was assessed using the independent classifications assigned by the three expert reviewers before consensus resolution. For each assay, electropherogram profiles were classified as polyclonal, oligoclonal, or monoclonal. Complete agreement was defined as identical classification by all three reviewers. Cases with any discordance among reviewers were submitted to consensus review and recorded as requiring consensus resolution. Interobserver reproducibility was quantified using percentage complete agreement and Fleiss’ kappa coefficients.

Only peaks located within the expected fragment-size ranges for each assay were considered for interpretation, according to the analytical design of each PCR system [7,35,36]. Peak dominance was evaluated in relation to the surrounding polyclonal background and to the overall electropherographic pattern, rather than as an isolated numerical value, in accordance with EuroClonality/BIOMED-2 interpretative principles [4,7]. Low-intensity, borderline, or poorly reproducible patterns were interpreted conservatively. When a restricted pattern was present but did not fulfill criteria for unequivocal monoclonality, the profile was classified as oligoclonal/intermediate. This conservative approach was adopted to reduce overclassification of borderline profiles as monoclonal, particularly in HTLV-1 carriers, in whom restricted or oligoclonal T-cell expansions may occur in non-neoplastic, virus-associated, or antigen-driven contexts [4,6,21,22,23].

### 2.7. Integrated Interpretative Workflow

In addition to the primary assay-specific interpretation, the study adopted an integrated interpretative workflow incorporating the results of TCRγ-A, TCRγ-B, and TCRγ-O. In the first step, TCRγ-A and TCRγ-B were interpreted together as complementary components of the EuroClonality/BIOMED-2 panel. This initial integrated assessment generated the following preliminary categories: non-clonal, when both assays showed polyclonal profiles; strong evidence of clonality, when both assays showed monoclonal profiles; weak evidence of clonality, when one assay showed a monoclonal profile and the other showed a polyclonal or oligoclonal profile; and oligoclonal expansion, when the combined EuroClonality profile consisted of polyclonal plus oligoclonal or oligoclonal plus oligoclonal results.

The terms “weak evidence of clonality” and “strong evidence of clonality” were author-defined interpretative categories created for the purposes of this workflow. They are not intended to represent universally accepted diagnostic classifications. Rather, they reflect the degree of concordance and reinforcement among the assays within the hierarchical algorithm. Therefore, these categories should be interpreted as structured molecular evidence levels requiring clinical correlation, not as stand-alone diagnostic labels.

**Figure 3:**
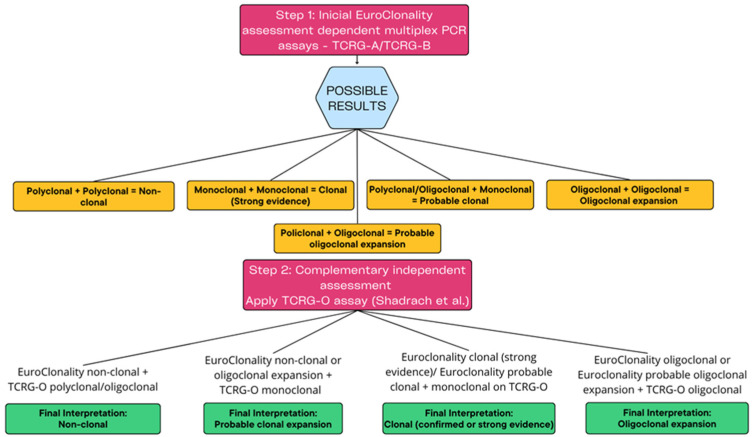
Integrated interpretative workflow for T-cell receptor gamma (TCRγ) clonality assessment based on the combined analysis of EuroClonality/BIOMED-2 T-cell receptor gamma assay A (TCRγ-A), EuroClonality/BIOMED-2 T-cell receptor gamma assay B (TCRγ-B), and the single-tube T-cell receptor gamma assay (TCRγ-O). In the first step, TCRγ-A and TCRγ-B were interpreted as complementary components of the EuroClonality/BIOMED-2 panel. In the second step, the result of the independent TCRγ-O assay was incorporated to refine the final classification into non-clonal, oligoclonal expansion, weak evidence of clonality, and strong evidence of clonality. Weak evidence of clonality and strong evidence of clonality are author defined workflow categories and do not represent universally established diagnostic classifications.

In the second step, the independent single-tube assay TCRγ-O was incorporated as a complementary interpretative layer. Cases initially classified as non-clonal by EuroClonality were finally interpreted as non-clonal when TCRγ-O showed a polyclonal or oligoclonal profile, and as weak evidence of clonality when TCRγ-O showed a monoclonal profile. Cases initially classified as strong evidence of clonality or weak evidence of clonality by EuroClonality were finally interpreted as strong evidence of clonality when TCRγ-O showed a monoclonal profile. Cases initially classified as oligoclonal expansion by EuroClonality were finally interpreted as oligoclonal expansion when TCRγ-O also showed an oligoclonal profile. This final classification was used for biological contextualization of the cases and for interpretation of the findings across the three study groups. A schematic representation of the workflow is shown in Figure 3.

### 2.8. Diagnostic Performance Analysis

For diagnostic performance analysis, clonality results were first classified into three primary analytical categories: polyclonal, oligoclonal, and monoclonal. Because oligoclonality represents a biologically and analytically distinct category rather than a simple classification error, oligoclonal profiles were not considered equivalent to either unequivocal polyclonality or unequivocal monoclonality in the primary interpretation [4,6,7,17,21,22,23,26,27]. Accordingly, three-category distributions were reported descriptively for all assays. For exploratory binary performance calculations, monoclonal results were considered positive and polyclonal results were considered negative, whereas oligoclonal results were handled as indeterminate/intermediate. A conservative sensitivity analysis was also performed in which oligoclonal results were classified as false positives in healthy donors and false negatives in ATLL patients, reflecting their limited discriminatory value in a strictly binary diagnostic framework. This analytical handling was restricted to diagnostic performance calculations and should not be interpreted as implying that oligoclonal profiles are biologically irrelevant. In HTLV-1 carriers, oligoclonality was analyzed separately as a potentially meaningful intermediate pattern.

Diagnostic performance calculations were restricted to healthy donors and ATLL patients, which were used as the reference negative and reference positive groups, respectively. HTLV-1 carriers were excluded from these calculations because of their recognized biological heterogeneity and the potential occurrence of intermediate clonality patterns in this group; they were therefore analyzed separately in a descriptive manner.

Because ATLL diagnosis in clinical practice relies on a composite diagnostic algorithm rather than on a single molecular assay, the reference positive group in this study was defined by established clinicopathological criteria, including Levine Diagnostic Criteria and Shimoyama classification [43,46]. The experimental TCRγ assays compared in the present study were not interpreted as isolated determinants of ATLL diagnosis. Nevertheless, because clonality assessment may contribute to the broader diagnostic work-up of ATLL, the possibility of incorporation bias cannot be entirely excluded. For this reason, diagnostic performance estimates were interpreted as exploratory apparent clinical-analytical performance within this cohort, rather than as fully independent diagnostic accuracy estimates.

For each assay, sensitivity, specificity, positive predictive value (PPV), and negative predictive value (NPV) were calculated according to standard definitions. In addition, a combined analysis of TCRγ-A+B was performed by considering a sample positive when monoclonality was detected in either of the two EuroClonality/BIOMED-2 tubes, in accordance with their complementary analytical design. Ninety-five percent confidence intervals for sensitivity and specificity were estimated using the Wilson method.

### 2.9. Reclassification and Statistical Analysis

After the primary analytical interpretation, a reclassification procedure based on the integrated interpretative workflow was performed to evaluate the effect of incorporating the independent TCRγ-O assay into the initial EuroClonality/BIOMED-2-based assessment. This step was intended to determine whether the addition of TCRγ-O modified the distribution of final classifications across the study groups and to assess the interpretative impact of the combined hierarchical approach. To compare the distribution of paired categorical classifications before and after application of the integrated workflow, the Stuart–Maxwell test was used [50,51]. This test was selected because it is appropriate for the evaluation of marginal homogeneity in correlated multinomial data and therefore allowed assessment of whether the incorporation of TCRγ-O significantly changed the classification profile obtained from the initial EuroClonality-based interpretation. All statistical analyses were performed using Python 3.11 and the IBM Statistical Package for the Social Sciences (SPSS) Statistics version 31, with standard statistical libraries.

To address paired comparisons among assays, McNemar’s exact tests were performed to assess whether the proportions of correct binary classifications differed between methods within the reference groups. Sensitivity comparisons were restricted to ATLL cases, whereas specificity comparisons were restricted to healthy donors. In addition, receiver operating characteristic (ROC) analyses were conducted using the clinically defined reference groups, with healthy donors coded as the reference negative group and ATLL patients as the reference positive group. For ordinal analyses, electropherogram categories were ranked according to increasing evidence of clonality. Areas under the ROC curve (AUCs) were estimated for each assay and compared using DeLong’s method when statistically estimable. When comparisons contained few or no discordant pairs, or when near-perfect separation limited AUC comparison, results were interpreted descriptively.

## 3. Results

### 3.1. Clinical and Biochemical Characterization of HTLV-1 Carriers and ATLL Patients

A total of 107 peripheral blood samples were included in the study, comprising 19 healthy donors, 75 HTLV-1 carriers, and 13 patients with adult T-cell leukemia/lymphoma (ATLL). For diagnostic performance analysis, only the reference groups were considered, namely healthy donors as the reference negative group and ATLL patients as the reference positive group, yielding a total of 32 samples. HTLV-1 carriers were analyzed separately because of their recognized biological heterogeneity and the expected occurrence of intermediate clonality patterns in this group. The mean age was 43.0 ± 11.2 years in the healthy donors group, 52.8 ± 12.6 years in the HTLV group, and 55.8 ± 16.3 years in the ATLL group. Males predominated in the control group (63.2%), whereas females were more frequent in both the HTLV carrier (82.7%) and ATLL (69.2%) groups. Among HTLV-infected individuals, vertical transmission was the most commonly reported route (64.0%), followed by sexual transmission (36.0%). Regarding clinical presentation in ATLL patients, the smoldering subtype was the most prevalent (46.2%), followed by the acute subtype (30.8%), chronic subtype (15.4%), and lymphoma subtype (7.7%). The demographic and clinical characteristics are summarized in Table 3.

**Figure 4:**
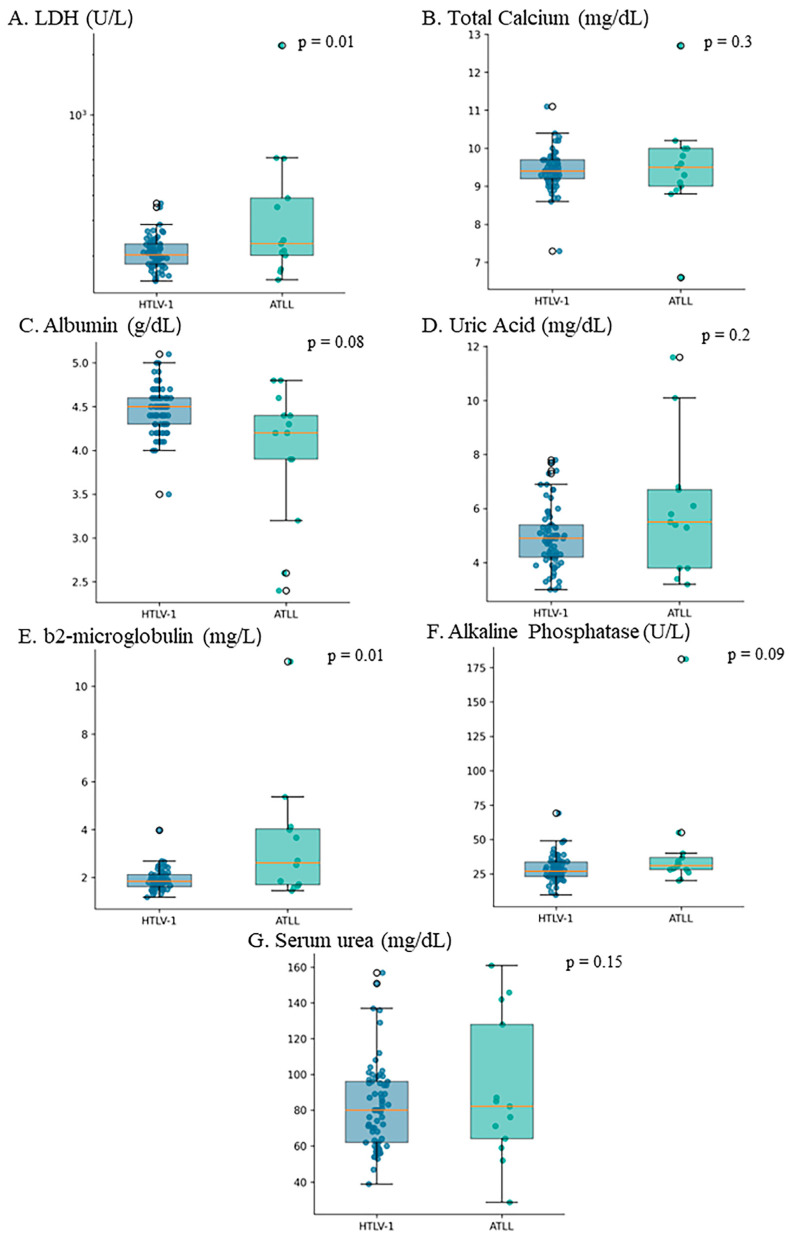
Graphical representation of the biochemical results in human T-lymphotropic virus type 1 (HTLV-1) carriers and patients with adult T-cell leukemia/lymphoma (ATLL). Boxplots with individual data points illustrate the distribution of (**A**) lactate dehydrogenase (LDH), (**B**) calcium, (**C**) albumin, (**D**) uric acid, (**E**) β2-microglobulin, (**F**) alkaline phosphatase, and (**G**) urea levels in HTLV-1 carriers and ATLL patients. Boxes represent the interquartile range (25–75th percentiles), central lines indicate the median, and whiskers indicate the data range. Individual points correspond to patient-level values. LDH is displayed on a logarithmic scale. Comparisons between groups were performed using the Mann–Whitney U test, with missing data excluded for each parameter. LDH and β2-microglobulin levels were significantly higher in ATLL patients, whereas calcium, albumin, uric acid, alkaline phosphatase, and urea showed no statistically significant differences between groups.

A total of 70/75 HTLV-1 carriers and 12/13 patients with ATLL were included in the biochemical analysis, with sample size varying according to data availability for each parameter. HTLV-1 carriers without available laboratory data for a given variable were excluded from the corresponding comparison. Biochemical parameters were compared between groups using the Mann–Whitney U test. Lactate dehydrogenase (LDH) levels were significantly higher in ATLL patients than in HTLV-1 carriers [median 239 units per liter U/L (25–75 percentile: 201–610) vs. 205 U/L (180–238); *p* < 0.01]. Similarly, β2-microglobulin levels were significantly elevated in the ATLL group [2.71 milligrams per liter (mg/L) (1.63–4.00) vs. 1.84 mg/L (1.60–2.18); *p* < 0.01]. In contrast, no statistically significant differences were observed for calcium [9.5 mg/dL (9.0–10.0) vs. 9.4 mg/dL (9.2–9.7); *p* = 0.30], albumin [4.2 g per deciliter (g/dL) (3.9–4.6) vs. 4.4 g/dL (4.2–4.7); *p* = 0.08], uric acid [5.4 mg/dL (3.8–6.8) vs. 4.9 mg/dL (4.3–5.6); *p* = 0.20], alkaline phosphatase [85 U/L (71–128) vs. 80 U/L (60–99); *p* = 0.09], or urea [31 mg/dL (28–40) vs. 28 mg/dL (24–34); *p* = 0.15]. Overall, ATLL patients showed higher LDH and β2-microglobulin levels, consistent with greater systemic disease burden compared with asymptomatic HTLV-1 carriers, as demonstrated in Figure 4.

### 3.2. Diagnostic Performance of Individual and Combined Assays in the Reference Groups

TCRγ-A and TCRγ-B showed identical sensitivity, with both assays correctly identifying 10 of 13 ATLL cases, corresponding to a sensitivity of 76.9%. Their specificities differed modestly, being 84.2% for TCRγ-A and 78.9% for TCRγ-B. Among the individual assays, TCRγ-O displayed the most conservative diagnostic profile, with the highest specificity (94.7%) and highest positive predictive value (88.9%), but the lowest sensitivity (61.5%), correctly identifying 8 of 13 ATLL cases while producing only one false-positive result among healthy donors. When the two EuroClonality/BIOMED-2 components were interpreted jointly, the combined TCRγ-A+B strategy yielded the highest sensitivity (100%), correctly identifying 13 of 13 ATLL cases, and the highest negative predictive value (100%). However, this gain in sensitivity was accompanied by lower specificity (73.7%), owing to an increased number of false-positive classifications among healthy donors. These findings demonstrate a clear analytical trade-off across methods. The combined EuroClonality strategy favored screening sensitivity, whereas TCRγ-O favored interpretative specificity. TCRγ-A and TCRγ-B, when considered individually, showed intermediate performance profiles; see Table 4.

Because the reference positive group included only 13 ATLL cases, all diagnostic performance estimates should be interpreted descriptively and with caution. The reported confidence intervals reflect the limited statistical precision associated with the small reference group and should not be interpreted as definitive diagnostic accuracy estimates.

Receiver operating characteristic (ROC) analysis was performed using healthy donors (HD) as the negative reference group and ATLL patients as the positive reference group. Clonality scores of 0, 0.5, and 1 were assigned to Non-clonal (NC), Oligoclonal expansion (OE), and Monoclonal profiles (ME), respectively. The TCRγ-O assay achieved an area under the curve (AUC) of 0.874, whereas TCRγ-A and TCRγ-B demonstrated higher discriminative performance, with AUC values of 0.949 and 0.943, respectively (Table 4). The combined TCRγ-A/B approach achieved an AUC of 1.000, indicating complete discrimination between ATLL patients and healthy donors. All ATLL samples were classified as ME by the combined approach, whereas no healthy donor sample reached the monoclonality category. DeLong comparisons among the three TCRγ assays showed no statistically significant differences (all *p* > 0.05). However, the relatively small sample size may have limited the statistical power to detect differences between ROC curves.

Pairwise comparisons of monoclonality detection were performed using McNemar’s exact test after dichotomization of results into monoclonal (ME) and non-clonal (NC/OE) categories, as described in Table S1. Among healthy donors, no significant differences were observed between any pair of assays, including the comparison between the original TCRγ-O assay and the classification obtained using the combined TCRγ-A/B interpretation strategy (all *p* = 1.000). Similarly, no significant differences were detected among ATLL patients (TCRγ-O vs. TCRγ-A, *p* = 0.688; TCRγ-O vs. TCRγ-B, *p* = 0.727; TCRγ-A vs. TCRγ-B, *p* = 1.000; TCRγ-O vs. TCRγ-A/B, *p* = 0.063).

In contrast, significant differences were identified within the HTLV-1 carrier group. Comparisons between TCRγ-O and TCRγ-B (*p* = 0.004) and between TCRγ-A and TCRγ-B (*p* = 0.012) reached statistical significance, whereas no difference was observed between TCRγ-O and TCRγ-A (*p* = 0.607). When the original TCRγ-O assay was compared with the final classification obtained using the combined TCRγ-A/B interpretation strategy, a significant difference was observed (*p* < 0.001), reflecting the identification of additional monoclonal cases through the integrated assessment of the assay’s gene fragments.

Overall, comparison between the original TCRγ-O assay and the final classification generated by the combined TCRγ-A/B interpretation strategy demonstrated a significant change in the distribution of monoclonal classifications when all samples were analyzed together (*p* < 0.001). This finding indicates that the integrated interpretation altered molecular classification patterns, particularly among HTLV-1 carriers. However, because the workflow was developed and evaluated within the same cohort, this reclassification effect should be interpreted as exploratory and should not be considered evidence of improved diagnostic accuracy without independent validation.

Interobserver agreement among the three evaluators was assessed using Fleiss’ kappa coefficient for clonal profile classification (polyclonal, oligoclonal, and monoclonal). The TCRγ-O assay demonstrated almost perfect agreement (κ = 0.825, 95% CI: 0.727–0.903), with complete agreement in 94/107 cases (87.9%) and 13 consensus cases. The TCRγ-A assay showed substantial agreement (κ = 0.737, 95% CI: 0.643–0.824), with complete agreement in 84/107 cases (78.5%) and 23 consensus cases. Similarly, the TCRγ-B assay exhibited almost perfect agreement (κ = 0.834, 95% CI: 0.750–0.907), with complete agreement in 92/107 cases (86.0%) and 15 consensus cases. When all TCRγ assays were analyzed collectively, the overall interobserver agreement remained high, reaching an almost perfect level (κ = 0.801, 95% CI: 0.748–0.848), with 270/321 cases (84.1%) showing complete agreement and 51 consensus cases across TCRγ-O, TCRγ-A, and TCRγ-B assays. These findings demonstrate the high reproducibility and consistency of clonal profile classification among independent evaluators across the different TCRγ assays, as shown in Table S2.

### 3.3. Assay-Specific Clonality Profiles in HTLV-1 Carriers

HTLV-1 carriers were analyzed separately because this group represented the most biologically heterogeneous context in the study. Across assays, clonality classification varied substantially, indicating that assay design influenced the detection and categorization of restricted T-cell expansions. TCRγ-B showed the highest rate of monoclonal classification, followed by TCRγ-A and TCRγ-O. Conversely, TCRγ-O yielded the highest proportion of polyclonal profiles, consistent with a more conservative classification profile. Oligoclonal patterns were most frequent in TCRγ-A and TCRγ-O, whereas TCRγ-B showed a lower frequency of such intermediate profiles; see Table 5.

These findings suggest that the assays differed not only in overall positivity rates but also in the way that biologically ambiguous cases were distributed across the three primary analytical categories. In particular, the higher proportion of monoclonal calls in TCRγ-B suggests greater analytical sensitivity for restricted clonal expansions, whereas the higher polyclonal rate observed with TCRγ-O is compatible with a more conservative interpretative profile. This variability provided the rationale for applying the integrated interpretative workflow.

### 3.4. Effect of the Integrated Interpretative Workflow by Study Group

The integrated interpretative workflow was then applied to determine whether incorporation of TCRγ-O modified the initial EuroClonality-based classification and improved biological discrimination across the study groups.

Among healthy donors (*n* = 19), 14 cases were initially classified as non-clonal and remained unchanged after incorporation of TCRγ-O. The remaining 5 cases, initially classified as possible oligoclonal, were reclassified as non-clonal after inclusion of TCRγ-O. No healthy donor case was reclassified into a category consistent with clonal expansion. These findings indicate that, in the non-neoplastic reference group, incorporation of TCRγ-O resolved borderline EuroClonality-based classifications toward a final non-clonal interpretation.

In the HTLV-1 carrier group (*n* = 75), the integrated workflow redistributed a substantial proportion of cases across final interpretative categories. Intermediate patterns identified by the initial EuroClonality-based assessment were variably reassigned after incorporation of TCRγ-O, with cases shifting toward non-clonal, oligoclonal expansion, weak evidence of clonality, or strong evidence of clonality according to the integrated algorithm. Overall, these findings indicate that the workflow did not simply increase or decrease positivity but redistributed borderline and intermediate patterns into more biologically informative final categories.

Among ATLL patients (*n* = 13), cases initially classified as strong evidence of clonality remained stable, whereas cases initially classified as weak evidence of clonality were predominantly reclassified as strong evidence of clonality after incorporation of TCRγ-O. No ATLL case was reclassified as non-clonal or oligoclonal expansion. Thus, in the neoplastic reference group, incorporation of TCRγ-O primarily reinforced clonal interpretation rather than qualitatively altering it, as described in Table 6.

### 3.5. Overall Reclassification Impact and Classification Stability

**Figure 5:**
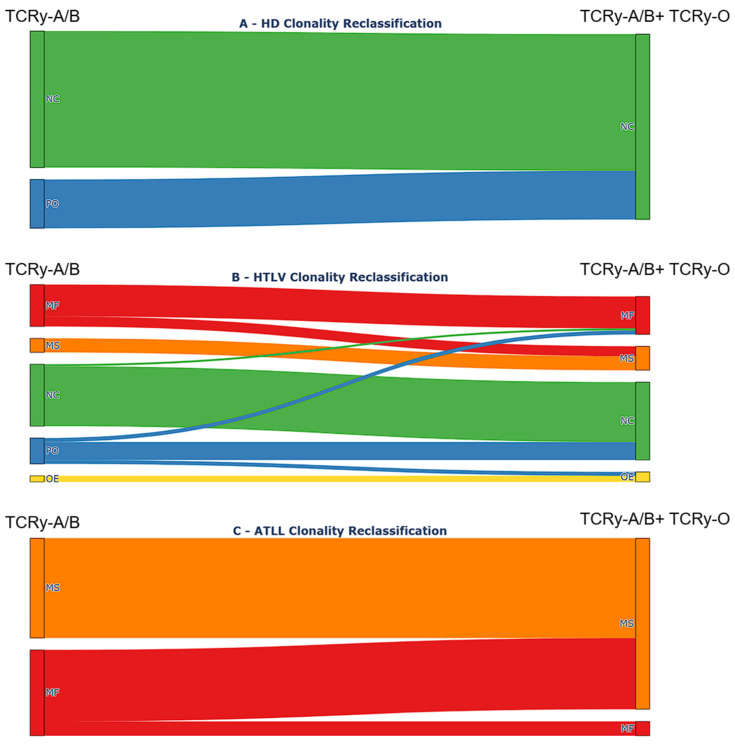
Sankey diagrams illustrating clonality reclassification following the combined analysis of TCRγ-A and TCRγ-B assays with the addition of the TCRγ-O assay. Sankey plots depict changes in clonality classification between the initial assessment based on TCRγ-A/B (left side) and the final classification obtained after incorporating TCRγ-O results (right side) in (**A**) healthy donors (HD), (**B**) human T-cell lymphotropic virus type 1 (HTLV-1) carriers, and (**C**) adult T-cell leukemia/lymphoma (ATLL) patients. The width of each flow is proportional to the number of samples undergoing a given classification transition. Clonality categories are represented as follows: NC, non-clonal (green); OE, oligoclonal expansion (yellow); PO, possible oligoclonal (blue); MS, strong evidence of clonality (orange); MF, weak evidence of clonality (red). Most samples retained their original classification after inclusion of the TCRγ-O assay, while a subset of HTLV-1 carriers exhibited reclassification between all categories. Weak evidence of clonality and strong evidence of clonality are author-defined workflow categories and do not represent universally established diagnostic classifications.

Overall, the application of the integrated workflow modified the classification of 21.8% of cases. The Stuart–Maxwell test demonstrated a statistically significant difference between the initial EuroClonality-based classification and the final classification after incorporation of TCRγ-O (*p* < 0.001), indicating that the reclassification effect was structured rather than random. The final integrated distribution of TCRγ clonality categories across healthy donors, HTLV-1 carriers, and ATLL patients is shown in Figure 5.

**Figure 6:**
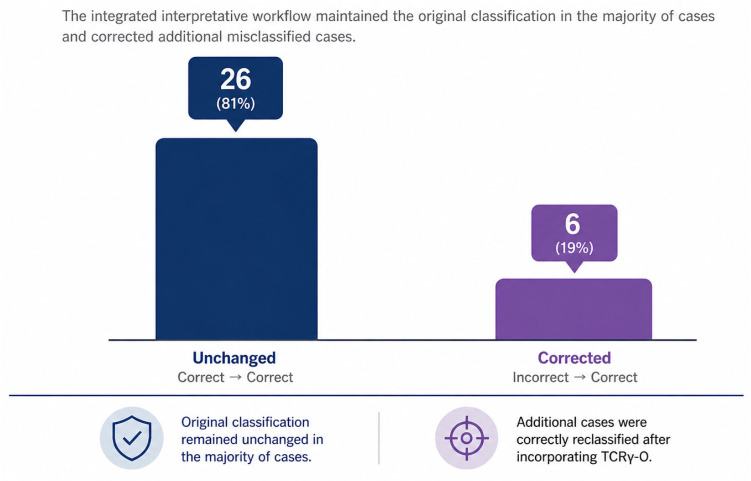
Effect of the integrated interpretative workflow on case classification in healthy donors and patients with adult T-cell leukemia/lymphoma (ATLL). The figure summarizes cases whose initial EuroClonality-based classification remained unchanged and cases that were reclassified after incorporation of the single-tube T-cell receptor gamma assay (TCRγ-O).

To further illustrate the practical effect of the integrated workflow on diagnostic interpretation, the stability and reclassification of cases in the reference groups are shown in Figure 6. Whereas the final classification profile in Figure 5 highlights the biological distribution of categories across the three study groups, Figure 6 emphasizes the direct impact of the algorithm on case-level interpretation, particularly the resolution of borderline classifications and the reinforcement of clonal assignment in ATLL.

## 4. Discussion

Molecular clonality testing is fundamentally an exercise in pattern interpretation, not a stand-alone disease label, and this principle is particularly relevant in studies of T-cell proliferations [52,53]. As emphasized by van Dongen et al. and later formalized by Langerek et al. [4,7], the analytical value of Ig/TCR clonality testing depends not only on primer design and technical execution, but also on disciplined interpretation within the appropriate clinical, morphologic, and immunophenotypic context [52,53]. In this setting, the present study addresses a practical and clinically important question: how different TCRγ assays behave across biologically distinct groups and whether their combined interpretation can improve the consistency of diagnostic classification.

Previous studies have demonstrated that HTLV-1 persistence is closely linked to the long-term expansion of infected T-cell clones and that ATLL usually emerges from a dominant malignant clone within a broader background of infected cells [20,23,41,54,55]. High-resolution methods, including HTLV-1 integration-site mapping and next-generation sequencing-based TCR repertoire analysis, have provided important insights into clonal architecture, clonal persistence, and early leukemogenic evolution [8,21,23,26,27,39,40,41]. Compared with these approaches, PCR-based TCRγ clonality testing provides lower clonal resolution, but remains more accessible and applicable to routine molecular diagnostic laboratories [6,7,36,38,39,56]. The present study should therefore be viewed as complementary to sequencing-based and integration-site approaches, offering a structured interpretative workflow for laboratories using capillary electrophoresis-based TCRγ analysis.

A central finding of the present work is that the three assays did not produce equivalent analytical profiles. The combined EuroClonality/BIOMED-2 strategy (TCRγ-A+B) showed the highest sensitivity, whereas TCRγ-O showed the highest specificity. This result is not only analytically relevant within the cohort but also analytically coherent. Because TCRγ-A and TCRγ-B were originally conceived as complementary tubes within the same EuroClonality system, their combined use is expected to maximize rearrangement coverage and reduce the likelihood of false-negative results, as already anticipated in the original BIOMED-2 design described by van Dongen et al. [7]. By contrast, a single-tube assay may provide a narrower but more conservative readout, which may explain the higher specificity observed for TCRγ-O in the present series.

The paired statistical comparisons further refine the interpretation of the apparent performance differences. Although the combined EuroClonality strategy and TCRγ-O showed different numerical profiles for sensitivity and specificity, McNemar’s exact test and AUC-based comparisons should be interpreted in light of the small number of discordant pairs in the reference groups. In practical terms, the high concordance observed among ATLL patients and healthy donors limits the ability of paired tests to demonstrate statistically significant superiority between assays, even when descriptive performance estimates differ. Therefore, the observed differences are best interpreted as complementary analytical tendencies within this exploratory cohort rather than as definitive evidence that one method is statistically superior to another.

This observation is consistent with the theoretical rationale of the proposed workflow. Because the TCRγ locus may undergo diverse Vγ-Jγ rearrangements, no single primer configuration is expected to capture all clonal architectures with identical efficiency [4,7,12]. The use of distinct but complementary primer designs increases the probability of detecting clonal populations that may be preferentially amplified in one reaction but less evident in another [4,7,36]. At the same time, concordance between assays strengthens interpretative confidence, whereas discordance may identify cases with low-level, borderline, oligoclonal, or technically complex patterns requiring cautious integrated interpretation [4,6,17]. Therefore, the advantage of the proposed strategy is not merely numerical improvement in sensitivity or specificity, but the creation of a biologically and technically informed interpretative hierarchy for TCRγ clonality assessment in HTLV-1 infection and ATLL.

These performance differences should not be framed as a simplistic competition between assays. Rather, they support the idea that the assays serve different analytical functions. In practical terms, the combined EuroClonality approach appears better suited to sensitive screening, whereas TCRγ-O appears more useful for specificity-oriented refinement, especially in samples with borderline or partially restricted patterns. This interpretation is consistent with the broader evolution of clonality testing discussed by van den Brand et al. [56], who highlighted that the clinical usefulness of clonality studies lies less in a binary “positive/negative” logic and more in how molecular findings are integrated into diagnostic reasoning.

The findings in healthy donors are particularly informative in this respect. In the non-neoplastic reference group, the integrated workflow resolved all borderline EuroClonality-based cases toward a final non-clonal interpretation and did not generate a final clonal classification in any healthy donor. This is diagnostically relevant because false-positive clonality interpretation is one of the most problematic outcomes in molecular hematopathology [57,58]. In other words, the addition of TCRγ-O did not merely add technical redundancy; it functioned as an orthogonal interpretative layer that helped stabilize the negative end of the biological spectrum. Such behavior is entirely compatible with the interpretative principles outlined by Langerek et al. [4], who stressed that clonality testing should support, rather than distort, the biological plausibility of the final diagnostic conclusion.

The additional interobserver agreement analysis supports the reproducibility of electropherogram interpretation in this study. Although capillary electrophoresis-based clonality analysis requires expert visual interpretation, the high percentage of complete agreement and the Fleiss’ kappa coefficients indicate that classification was not dependent on a single subjective reading. Nevertheless, cases requiring consensus highlight the importance of standardized interpretation criteria, particularly for borderline or oligoclonal profiles.

At the opposite end of the spectrum, the ATLL group behaved as expected for a mature T-cell neoplasm associated with dominant clonal expansion. Cases already classified as strong evidence of clonality remained stable, whereas those initially classified as weak evidence of clonality were predominantly shifted toward stronger clonal interpretation after incorporation of TCRγ-O. This finding suggests that the orthogonal assay did not destabilize clearly neoplastic cases; rather, it reinforced clonal interpretation in samples already biologically expected to harbor dominant T-cell clones. This is in line with current hematolymphoid classification frameworks, including the WHO-based approach summarized by Karube et al. [1,33], in which ATLL is recognized as a mature T-cell neoplasm with a clinicopathologic and molecular context strongly favoring clonal dominance.

The most analytically challenging and biologically revealing findings emerged in HTLV-1 carriers. This group showed the broadest inter-assay variability, with TCRγ-B generating the highest rate of monoclonal classifications and TCRγ-O the highest rate of polyclonal classifications. Such divergence is biologically plausible. HTLV-1 establishes persistent infection in T cells and drives long-term expansion of infected clones, creating a continuum that may range from broad polyclonality to marked clonal skewing [55,59,60,61]. As reviewed by Clauze et al. [21], T-cell repertoire behavior in HTLV-1-associated disease is dynamic and heterogeneous, and intermediate clonal structures are not only possible but expected. Therefore, the discordance observed between assays in the HTLV-1 carrier group should not be interpreted simply as technical inconsistency; it likely reflects the underlying complexity of the clonal landscape being measured.

This point becomes even more relevant when considered alongside prior evidence that clonality in HTLV-1 infection may have prognostic significance [23,62,63]. Firouzi et al. [23] showed that the clonality of HTLV-1-infected cells may serve as a risk indicator for the development and progression of adult T-cell leukemia, suggesting that clonal architecture is not merely descriptive but potentially informative for disease evolution. The present study did not assess viral integration-site clonality and does not claim prognostic validation. Nevertheless, its findings are conceptually aligned with that literature: the very group expected to harbor biologically intermediate and evolving clonal structures was the group in which assay behavior was most divergent and in which an integrated workflow proved most interpretatively useful [23].

In this context, the integrated interpretative workflow represents the main methodological and exploratory contribution of the study. The significant reclassification effect demonstrated by the Stuart–Maxwell test indicates that the workflow altered classification in a structured and non-random way. More importantly, the direction of reclassification was biologically coherent: healthy donors concentrated in the non-clonal category, ATLL cases in strong evidence of clonality, and HTLV-1 carriers across the intermediate interpretative space. This gradient argues against arbitrary reshuffling and supports the view that the workflow is capturing biologically meaningful variation rather than merely adding another layer of molecular complexity.

A further strength of the study is that it preserves the conceptual integrity of the EuroClonality system while acknowledging the value of orthogonal confirmation. The manuscript does not treat TCRγ-A and TCRγ-B as two independent competing methods, which would be a methodological misreading of the BIOMED-2 framework. Instead, it respects their intended design as complementary reactions and then asks whether an additional assay can improve final interpretation. This is a more mature diagnostic strategy, because in real-world practice the relevant question is rarely “which assay is superior in isolation?” but rather “how can partially overlapping molecular signals be integrated to reduce uncertainty?” This orientation is highly consistent with the standardized interpretative philosophy proposed by Langerek et al. [4].

The study also provides a potential practical perspective for molecular diagnostics, although routine implementation would require independent validation. In laboratories already using EuroClonality-based testing, the current data suggest that a hierarchical interpretative model may help organize assay-specific findings, particularly in borderline or biologically heterogeneous samples. This should not be interpreted as a proposal for immediate clinical implementation, but rather as an exploratory framework for future validation. In this regard, the study fits well within the broader diagnostic evolution described by van den Brand et al. [56], in which the goal of clonality testing is not only detection but also improved integration of molecular results into real diagnostic workflows.

Another notable strength is the choice of study groups. By including healthy donors, HTLV-1 carriers, and ATLL patients, the study did not restrict itself to a simple case–control comparison between unequivocally negative and unequivocally positive states. Instead, it deliberately incorporated the biologically intermediate space in which diagnostic uncertainty is greatest. This design is particularly valuable because many clonality studies perform best at the extremes but provide less insight into the settings where interpretation is most difficult. Here, the inclusion of HTLV-1 carriers substantially increases the translational relevance of the findings, precisely because that group represents the setting in which assay complementarity matters most.

The study must, however, be interpreted in light of important limitations. First, the number of ATLL cases is small. This limitation is particularly relevant for the interpretation of diagnostic performance estimates because small sample sizes reduce statistical precision, widen confidence intervals, and increase the instability of sensitivity, specificity, positive predictive value, and negative predictive value estimates. Nevertheless, this limitation should be considered in the epidemiological context of ATLL. ATLL is a rare HTLV-1-associated mature T-cell neoplasm that develops in only a minority of infected individuals, usually after prolonged viral persistence and latency [1,20,22,33,54,64]. Therefore, the inclusion of 13 clinically characterized ATLL cases over a three-year recruitment period represents a meaningful and clinically relevant series for an exploratory single-center molecular workflow study. Accordingly, the performance measures reported here should be interpreted as exploratory estimates of apparent clinical analytical behavior within this cohort, rather than as definitive universal diagnostic accuracy parameters. Replication in larger, preferably multicenter cohorts will be necessary before generalizing these values more broadly.

Second, the study lacks an independent molecular gold standard for adjudicating assay discordance. The assays were compared against clinically defined reference groups rather than against sequencing-based TCR clonality, integration-site mapping, or another orthogonal molecular method. This is acceptable for a clinically oriented study, but it imposes an important interpretative limit: when two assays disagree, the present design cannot determine with certainty which assay is molecularly correct. This limitation is especially relevant for HTLV-1 carriers with oligoclonal or weakly clonal profiles [65,66], in whom conventional PCR-based clonality testing cannot determine whether the observed pattern represents reactive antigen-driven expansion, virus-associated clonal persistence, early premalignant selection, or an emerging malignant clone. Future validation against higher-resolution methods will therefore be essential.

Third, oligoclonal profiles remain intrinsically difficult to handle. In this study, they were treated as analytically ambiguous events for the purpose of diagnostic performance calculations, which is methodologically defensible because it preserves separation between unequivocally non-neoplastic and neoplastic reference groups. At the same time, oligoclonality is not necessarily just interpretative noise. In chronic antigenic stimulation and virus-associated clonal proliferation, it may reflect a biologically meaningful intermediate state [19,27,67,68]. Thus, while the present handling of oligoclonal patterns is appropriate for performance analysis, future work should examine these profiles not only as classification problems but also as potential indicators of clonal instability or evolving disease biology. This is particularly relevant in HTLV-1 infection [27,55,69]. Clinically, this means that oligoclonal profiles in HTLV-1 carriers should not be interpreted as diagnostic of ATLL in isolation, but rather as findings that may justify closer clinical correlation, immunophenotypic assessment, proviral load evaluation when available, and longitudinal monitoring. Therefore, the integrated workflow treats oligoclonal expansion as a distinct interpretative category, and binary performance metrics should be understood as simplified exploratory summaries rather than complete biological classifications.

Another limitation is that the proposed workflow, although biologically coherent and statistically associated with significant reclassification, was developed and evaluated in the same cohort. Therefore, the observed improvement in classification may partly reflect fit to the present study population rather than generalizable performance. No independent internal validation, cross-validation, or external validation was performed. The results should therefore be interpreted as the apparent performance of a rule-based exploratory algorithm. Future studies should evaluate the workflow in independent cohorts, preferably using prospective multicenter designs, prespecified interpretative rules, interobserver reproducibility assessment, and orthogonal molecular approaches such as TCR next-generation sequencing or HTLV-1 integration-site analysis [8,21,23,27,39,40,41].

The results also point toward several concrete future directions. One is validation in larger and longitudinal cohorts of HTLV-1 carriers, which would make it possible to test whether final categories such as weak evidence of clonality or oligoclonal expansion correlate with future clinical evolution, proviral load, or progression to ATLL [70,71,72]. Another is integration with higher-resolution molecular methods, including next-generation sequencing (NGS) based TCR clonality assays [8,73,74,75]. Recent comparative work by Donelli et al. (2024) indicates that sequencing-based approaches may improve clonal resolution, although conventional PCR-based methods remain highly relevant in current practice [12]. A third important direction would be HTLV-1 integration-site or infected-clone profiling, which could help clarify whether discordant molecular patterns reflect true low-level clonal populations, assay-specific blind spots, or biologically unstable repertoires [76,77,78,79].

Modern high-resolution approaches may overcome some of the limitations of conventional PCR-based clonality testing. Next-generation sequencing-based TCR repertoire analysis can identify and quantify clonotypes with higher resolution, distinguish dominant clones from complex oligoclonal backgrounds, and support longitudinal monitoring of clonal dynamics [8,21,39,41]. In HTLV-1 infection, proviral integration-site mapping provides an additional layer of biological information by identifying infected cellular clones and estimating their relative abundance over time [22,23,26,27,41,54]. These approaches may be particularly valuable for HTLV-1 carriers with oligoclonal or weakly clonal profiles, in whom conventional capillary electrophoresis cannot determine whether restricted patterns represent reactive antigen-driven expansion, persistent infected clones, premalignant clonal selection, or early malignant transformation. Nevertheless, conventional PCR-based TCRγ testing remains more accessible in many diagnostic laboratories and may still provide clinically useful information when interpreted within a structured and conservative workflow [4,6,7,36,37,38,39]. The proposed workflow should therefore be viewed as complementary to, rather than as a replacement for, high-resolution sequencing and integration-site approaches.

Overall, the present data support a diagnostic model in which TCRγ clonality assays are best understood as complementary analytical tools rather than interchangeable competitors. The combined EuroClonality approach appears best suited for sensitive screening, whereas TCRγ-O appears especially useful as a specificity-oriented orthogonal assay. This complementarity becomes most visible in HTLV-1 carriers, the main group in which biological heterogeneity makes interpretation most difficult and most clinically relevant.

By organizing assay outputs into a hierarchical interpretative workflow, the study proposes a biologically grounded exploratory strategy for structuring TCRγ clonality interpretation. Its potential utility in routine molecular hematopathology should be evaluated in larger independent cohorts before clinical implementation. These findings highlight how molecular clonality testing, when interpreted through a structured and hierarchical workflow, may expand the practical contribution of laboratory medicine in the era of genetic testing, particularly in biologically heterogeneous settings such as chronic HTLV-1 infection.

## 5. Conclusions

This study demonstrates that TCRγ clonality assays based on EuroClonality/BIOMED-2 and a complementary single-tube multiplex PCR assay provide distinct and analytically informative profiles. The combined EuroClonality strategy achieved the highest sensitivity, whereas TCRγ-O achieved the highest specificity, supporting a complementary rather than competitive interpretation of these methods.

The integrated interpretative workflow significantly altered classification patterns and provided a structured description of biologically distinct groups among healthy donors, HTLV-1 asymptomatic carriers, and ATLL patients. These findings support a hierarchical approach in which EuroClonality-based assays maximize screening sensitivity while TCRγ-O contributes additional interpretative specificity, particularly in the biologically heterogeneous context of HTLV-1 infection. However, because the workflow was developed and evaluated within the same cohort, included a limited number of ATLL cases, and lacks independent external validation, it should be considered an exploratory research tool and not a ready-to-use diagnostic algorithm for routine clinical practice. The observed improvements in monoclonality detection and classification should therefore be interpreted as preliminary and hypothesis-generating rather than as evidence of established diagnostic superiority or improved diagnostic accuracy. Future multicenter studies incorporating longitudinal follow-up and orthogonal molecular methods, including TCR next-generation sequencing and HTLV-1 integration-site analysis, are required to determine the reproducibility, generalizability, and clinical utility of this approach.

## Figures and Tables

**Table 1:** Single-tube T-cell receptor gamma assay (TCRγ-O) primer sequences for amplification of the variable and joining (VJ) region gene gamma fragments, with respective probes, amplification cycles, and band analysis.

Primers of TCRγ-O	Sequences (5′–3′)	5′ Probe	Amplification Cycles	Band Analysis	References
Vγ (1–8)	ACCAGGAGGGGAAGGCCCCACAG	FAM	3 min 94 °C; 1 min 95 °C, 30 s 61.8 °C, 30 s 72 °C (40×); 10 min 72 °C; ∞ 4 °C	FAM/HEX 150 to 250 bp	Shadrach and Warshawsky, 2004 [35]
Vγ9	AGAAAGGAATCTGGCATTCCG	FAM			
Vγ10	AATCCGCAGCTCGACGCAGCA	FAM			
Vγ11	GCTCAAGATTGCTCAGGTGGG	HEX			
Jγ1/2	ACCTGTGACAACAAGTGTTGTTC	-			
JγP1/2	AGTTACTATGAGC(T/C)TAGTCCC	-			
JγP	TGTAATGATAAGCTTTGTTCC	-			

Legend: TCRγ-O, single-tube T-cell receptor gamma assay, Shadrach methodology; Vγ, variable gamma region; Jγ, joining gamma region; FAM, 6-carboxyfluorescein; HEX, hexachloro-fluorescein; bp, base pair; A, adenine; T, thymine; C, cytosine; G, guanine; min, minutes; s, seconds; °C, Celsius degree; ∞, infinity.

**Table 2:** T-cell receptor gamma assay A (TCRγ-A) and T-cell receptor gamma assay B (TCRγ-B) (EuroClonality/BIOMED-2) primer sequences for amplification of variable and joining (VJ) region gene gamma fragments, with respective probes, amplification cycles, and band analysis.

Primers of TCRγ-A and TCRγ-B	Sequences (5′–3′)	5′ Probe	Amplification Cycle	Band Analysis	References
TCRγ-A-Vγ1f	GGAAGGCCCCACAGCRTCTT	-	5 min 95 °C; 45 s 95 °C, 45 s 60 °C, 45 s 72 °C (40×); 10 min 72 °C; ∞ 4 °C	TCRγ-A: FAM 145 to 175 and 195 to 230; HEX 175 to 195 and 230 to 255 bp. TCRγ-B: FAM 80 to 110 and 145 to 170; HEX 110 to 145 and 170 to 210.	van Dongen et al., 2003 [7] * This study
TCRγ-A-Vγ10	AGCATGGGTAAGACAAGCAA	-			
TCRγ-A/B-Jγ1.1/2.1 (JP1/2)	TTACCAGGCGAAGTTACTATGAGC	HEX *			
TCRγ-A/B-Jγ1.3/2.3 (Jγ1/2)	GTGTTGTTCCACTGCCAAAGAG	FAM *			
TCRγ-B-Vγ9	CGGCACTGTCAGAAAGGAATC	-			
TCRγ-B-Vγ11	CTTCCACTTCCACTTTGAAA	-			

Legend: TCRγ-A/B, T-cell receptor gamma assays A and B, van Dongen methodology; Vγ, variable gamma region; Jγ, joining gamma region; FAM, 6-carboxyfluorescein; HEX, hexachloro-fluorescein; bp, base pair; A, adenine; G, guanine; T, thymine; C, cytosine; min, minutes; s, seconds; °C, Celsius degree; ∞, infinity; *, Localization of 5’ probes designed by this study.

**Table 3:** Demographic and clinical characteristics of the study population.

Variable	HD (*n* = 19)	HTLV-1 (*n* = 75)	ATLL (*n* = 13)
**Age (years)**			
Mean ± SD	43.0 ± 11.2	52.8 ± 12.6	55.8 ± 16.3
Median (IQR)	41 (33–51.5)	53 (45–61)	55 (43–67)
Min–Max	26–61	19–76	27–83
**Sex, *n* (%)**			
Male	12 (63.2)	13 (17.3)	4 (30.8)
Female	7 (36.8)	62 (82.7)	9 (69.2)
**Transmission route, *n* (%)**			
Vertical	—	48 (64.0)	—
Sexual	—	27 (36.0)	—
**ATLL subtype, *n* (%)**			
Smoldering	—	—	6 (46.2)
Acute	—	—	4 (30.8)
Chronic	—	—	2 (15.4)
Lymphoma	—	—	1 (7.7)

Legend: HD, healthy donors; HTLV-1, human T-lymphotropic virus type 1 carriers; ATLL, Adult T Cell Leukemia/Lymphoma patients; SD, standard deviation; IQR, interquartile range; min, minimum; max, maximum; *n*, number; %, percentage.

**Table 4:** Diagnostic performance of individual and combined T-cell receptor gamma (TCRγ) clonality assays in healthy donors and patients with adult T-cell leukemia/lymphoma (ATLL).

Assay	TP	FN	FP	TN	Sensitivity % (95% CI)	Specificity % (95% CI)	PPV %	NPV %	ROC Curve
TCRγ-A	10	3	3	16	76.9 (49.7–91.8)	84.2 (62.4–94.5)	76.9	84.2	0.949
TCRγ-B	10	3	4	15	76.9 (49.7–91.8)	78.9 (56.7–91.5)	71.4	83.3	0.943
TCRγ-O	8	5	1	18	61.5 (35.5–82.3)	94.7 (75.4–99.1)	88.9	78.3	0.874
EuroClonality (A+B)	13	0	5	14	100.0 (77.2–100.0)	73.7 (51.2–88.2)	72.2	100.0	1.000

Legend: TP, true positives; FN, false negatives; FP, false positives; TN, true negatives; PPV, positive predictive value; NPV, negative predictive value; CI, confidence interval; ROC curve, Receiver Operating Characteristic.

**Table 5:** Distribution of primary clonality categories across TCRγ assays in HTLV-1 carriers.

Assay	Polyclonal, *n* (%)	Oligoclonal, *n* (%)	Monoclonal, *n* (%)
**TCRγ-A**	41 (54.7)	18 (24.0)	16 (21.3)
**TCRγ-B**	40 (53.3)	11 (14.7)	24 (32.0)
**TCRγ-O**	47 (62.7)	16 (21.3)	12 (16.0)

Legend: TCRγ-A/B, T cell receptor gamma tubes A and B (Euroclonality/BIOMED-2); TCRγ-O, single-tube T-cell receptor gamma assay tube O; *n*, number; %, percentage.

**Table 6:** Reclassification of cases after application of the integrated interpretative workflow, according to the study group.

Study Group	Initial Classification	Final Classification	*n*
**Healthy donors**	Non-clonal	Non-clonal	14
**Healthy donors**	Possible oligoclonal	Non-clonal	5
**HTLV-1 carriers**	Non-clonal	Non-clonal	30
**HTLV-1 carriers**	Non-clonal	Weak evidence of clonality	1
**HTLV-1 carriers**	Possible oligoclonal	Non-clonal	9
**HTLV-1 carriers**	Possible oligoclonal	Weak evidence of clonality	2
**HTLV-1 carriers**	Possible oligoclonal	Oligoclonal expansion	5
**HTLV-1 carriers**	Weak evidence of clonality	Weak evidence of clonality	16
**HTLV-1 carriers**	Weak evidence of clonality	Strong evidence of clonality	5
**HTLV-1 carriers**	Strong evidence of clonality	Strong evidence of clonality	7
**ATLL**	Strong evidence of clonality	Strong evidence of clonality	7
**ATLL**	Weak evidence of clonality	Strong evidence of clonality	5
**ATLL**	Weak evidence of clonality	Weak evidence of clonality	1

Legend: HTLV-1, human T-lymphotropic virus type 1; ATLL, adult T-cell leukemia/lymphoma; *n*, number.

## Data Availability

The data supporting the findings of this study are available from the corresponding author upon reasonable request. Due to ethical and privacy restrictions involving human-derived samples, the data are not publicly available.

## Supplementary Materials

The following supporting information can be downloaded at: https://www.mdpi.com/article/10.3390/diagnostics16142288/s1, Table S1. Pairwise comparisons of monoclonality detection using McNemar’s exact test after dichotomization into monoclonal (ME) and non-clonal (NC/OE) categories; Table S2. Interobserver agreement among three evaluators for 321 clonal profile classifications.

## Abbreviations

The following abbreviations are used in this manuscript:

ANVISA	Agência Nacional de Vigilância Sanitária
ATLL	Adult T-cell leukemia/lymphoma
BIOMED-2	BIOMED-2 Concerted Action
bp	Base pairs
CAAE	Certificado de Apresentação de Apreciação Ética
CAPES	Coordenação de Aperfeiçoamento de Pessoal de Nível Superior
CD4	Cluster of differentiation 4
CI	Confidence interval
CNPq	Conselho Nacional de Desenvolvimento Científico e Tecnológico
DNA	Deoxyribonucleic acid
dNTPs	Deoxynucleotide triphosphates
EDTA	Ethylenediaminetetraacetic acid
FAM	6-carboxyfluorescein
FN	False negatives
FP	False positives
HD	Healthy donors
FM-USP	Faculdade de Medicina da Universidade de São Paulo
HCFMUSP	Hospital das Clínicas, Faculdade de Medicina, Universidade de São Paulo
HEX	Hexachloro-fluorescein
HTLV-1	Human T-lymphotropic virus type 1
Ig	Immunoglobulin
IMT	Instituto de Medicina Tropical
IQR	Interquartile range
Jγ	Joining gamma region
LDH	Lactate dehydrogenase
LIM-31	Laboratory of Pathogenesis and Directed Therapy in Onco-Immuno-Hematology
NCBI	National Center for Biotechnology Information
NGS	Next-generation sequencing
NPV	Negative predictive value
PBMCs	Peripheral blood mononuclear cells
PCR	Polymerase chain reaction
PPV	Positive predictive value
SD	Standard deviation
SPSS	Statistical Package for the Social Sciences
TCR	T-cell receptor
TCRγ	T-cell receptor gamma
TCRγ-A	EuroClonality/BIOMED-2 T-cell receptor gamma assay A
TCRγ-B	EuroClonality/BIOMED-2 T-cell receptor gamma assay B
TCRγ-O	Single-tube T-cell receptor gamma assay
TN	True negatives
TP	True positives
TRG	T-cell receptor gamma gene
USP	University of São Paulo
V(D)J	Variable, diversity, and joining
Vγ	Variable gamma region
WHO	World Health Organization
