# Supplementary material for: Integrated TCRγ Clonality Workflow for Molecular Diagnosis in HTLV-1 Carriers and Adult T-Cell Leukemia/Lymphoma
Source: Diagnostics (Basel). 2026 Jul 22;16(14):2288. doi: 10.3390/diagnostics16142288 (PMC13407627; doi:10.3390/diagnostics16142288)
Supplement: Supplementary file 1 [file diagnostics-16-02288-s001.zip › diagnostics-4341711-supplementary.pdf]

Table S1. Pairwise comparisons of monoclonality detection using McNemar's exact test after dichotomization into monoclonal (ME) and non-clonal (NC/OE) categories.

Comparison	Healthy Donors (p value)	HTLV-1 Carriers (p value)	ATLL (p value)
TCRγ-O vs TCRγ-A	1.000	0.607	0.688
TCRγ-O vs TCRγ-B	1.000	0.004*	0.727
TCRγ-A vs TCRγ-B	1.000	0.012*	1.000
TCRγ-O vs Integrated TCRγ-A/B interpretation	1.000	<0.001*	0.063

Legend: TCRγ-A/B, T-cell receptor gamma assays A and B, van Dongen methodology; TCRγ-O, single-tube T-cell receptor gamma assay, Shadrach methodology T-cell receptor gamma assay O.

*Footnote: Pairwise comparisons were performed using McNemar's test after dichotomization of clonality results into monoclonal and non-monoclonal categories. P-values indicate whether significant differences existed in monoclonality classification between assays within each study group. Statistical significance was defined as $p < 0.05$ and is indicated by an asterisk (*).

Table S2. Interobserver agreement among three evaluators for 321 clonal profile classifications.

Variable	Number of cases	Complete agreement (%)	Consensus review required, n (%)	Statistic	Value	95% CI	Interpretation
TCRγ-O clonal profile classification	107	94/107 (87.9%)	13/107 (12.1%)	Fleiss' kappa (κ)	0.825	0.727–0.903	Almost perfect agreement
TCRγ-A clonal profile classification	107	84/107 (78.5%)	23/107 (21.5%)	Fleiss' kappa (κ)	0.737	0.643–0.824	Substantial agreement
TCRγ-B clonal profile classification	107	92/107 (86.0%)	15/107 (14.0%)	Fleiss' kappa (κ)	0.834	0.750–0.907	Almost perfect agreement
Overall clonal profile classification	321*	270/321 (84.1%)	51/321 (15.9%)	Fleiss' kappa (κ)	0.801	0.748–0.848	Almost perfect agreement

Legend: CI, confidence interval. TCRγ-A/B, T-cell receptor gamma assays A and B, van Dongen methodology; TCRγ-O, single-tube T-cell receptor gamma assay, Shadrach methodology T-cell receptor gamma assay O; κ, Kappa value.
